# Advanced Vibration-Based Fault Diagnosis and Vibration Control Methods

**DOI:** 10.3390/s23187704

**Published:** 2023-09-06

**Authors:** Xiaohua Song, Jing Liu, Min Xia

**Affiliations:** 1 School of Electronic Information Engineering, Xi’an Technological University, Xi’an 710021, China; sxh831018@163.com; 2 School of Marine Science and Technology, Northwestern Polytechnical University, Xi’an 710072, China; 3 Department of Engineering, Lancaster University, Lancaster LA1 4YW, UK; m.xia3@lancaster.ac.uk

## 1. Introduction

Fault diagnosis and vibration control are the tracking of any aspect of an industry mechanical components’ performance using reliably measured data and analytical simulations in conjunction with the heuristic experience, so that the current and expected future performance of the machine for at least the most critical limit events can be described in a proactive manner. Advanced intelligent fault diagnosis methods, together with abundant measurement and simulation information, can greatly improve the performances of various industry mechanical systems. Although a lot of works have been introduced to develop methods for determining the working status of different machines using different detection techniques [1], it is still challenging to accurately determine the time-varying working status, because practical working conditions are extremely complex and stochastic [2]. Classical statistical features obtained from the raw vibration signal fail to hold the understanding and steadiness required for fault detection, exclusively in multifaceted noisy situations [3]. The proper application and integration of big data analytics, signal processing methods, measurement systems, fast computing, and machine learning can lead to the development of accurate diagnosis and prognosis methods for mechanical systems in industry applications. Moreover, advanced vibration control methods can be helpful for preventing the failures of mechanical components.

Despite a lot of advancement in the field of fault diagnosis and vibration control methods, the advanced industry faces the following challenges while developing accurate fault diagnosis solutions. There are limited labelled data about machine health. The developed methods work satisfactorily on the machines upon which training is performed. However, when working conditions are introduced, such as complex loading and operating speed conditions, theoretical methods have a low accuracy. The most important issue is that the industrial environment does not allow for many investigations.

In order to rapidly report and spread the latest advancements in the science of fault diagnosis, including new discoveries and valuable applied investigations from all over the world, this Special Issue’s main aim is to gather state-of-the-art research and review articles into all aspects of theoretical and applied investigations about the latest developments in new sensor technologies and diagnosis methods for the detection of faults. The topics include vibration-based fault diagnosis; new sensor technologies; vibration control methods; the failure mechanism of the faults in various mechanical systems; the vibro-acoustic diagnosis of machinery; and case studies and industrial applications.

## 2. Overview of the Contributions

This Special Issue on “Advanced Vibration-Based Fault Diagnosis and Vibration Control Methods” includes twelve papers ranging across different methods used in the electrical and mechanical industries’ systems. The papers include a good literature review of the current new works on the verification of mechanical properties identification based on the impulse excitation technique and mobile device measurements [4], the method for bearing fault diagnosis under variable speeds based on envelope spectrum fault characteristic frequency band identification [5], the interface design of head-worn display applications for condition monitoring in aviation [6], color recurrence plots for bearing fault diagnoses [7], the failure mode detection and validation of a shaft-bearing system with common sensors [8], the strain response and buckling behavior of composite cylindrical shells subjected to external pressure with one end fixed and the other free of boundary conditions [9], the smartphone-based and data-driven superstructure state prediction method for highway bridges in service [10], the optimal control algorithm for stochastic systems with parameter drift [11], the nonlinear vibration control experimental system design of a flexible arm using interactive actuations from shape memory alloy [12], the design of an active vibration isolation controller with a disturbance observer-based linear quadratic regulator for optical reference cavities [13], the applicability of touchscreens in manned/unmanned aerial vehicle cooperative missions [14], and the engineering frequency domain analysis and vibration suppression of flexible aircraft based on an active disturbance rejection controller [15].

The Editorial paper is a brief summary of the content of the 12 papers published in the Sensors Special Issue “Advanced Vibration-Based Fault Diagnosis and Vibration Control Methods”. A brief summary of the content associated with each of the chosen papers belonging to the Special Issue is given as follows:

Advanced, vibration-based fault diagnosis and vibration control methods for mechanical and electrical systems should be useful for condition monitoring and vibration control designs. Many works have been reported to present various studies on effective detection and vibration control.

The Impulse Excitation Technique (IET) is a useful testing method for calculating or evaluating some material properties. However, it is a requirement for specialized sensor and acquisition systems and well-trained engineers to prepare the setup and analyze the results. Thus, Scislo [4] evaluated the possibility of a low-cost solution in the form of a mobile device microphone as a way of obtaining the data. By using Fast Fourier Transform, it allows frequency response graphs to be obtained and uses the IET method procedure to calculate the mechanical properties of samples. Moreover, this method does not require specific knowledge of sensing technology, signal treatment, or data analysis. It can be performed by any assigned employee, who can receive the quality check information immediately on-site. The presented procedure also allows for data collection and transfer to the cloud for future reference and additional information extraction.

The vibration signals of rolling element bearings with variable speeds have nonstationary characteristics. Order tracking and time–frequency analysis are two widely methods for the fault diagnosis of these rolling element bearings with variable speeds. However, the order tracking method could be influenced by close-order harmonic interference and resampling errors; moreover, the accuracy of the time–frequency analysis method is mainly limited by ridge extraction algorithms and time–frequency resolutions. To address this problem, Pei et al. [5] proposed a method based on envelope spectrum fault characteristic frequency band identification. In this method, the envelope spectrum of the fault vibration signals of the bearings under variable speeds are analyzed, and the fault characteristic frequency band is introduced as a new and effective representation of faults. The fault templates according to the fault characteristic frequency band are constructed as a reference for the fault identification. By using the correlation coefficients between the envelope spectrum and the fault templates in the extended fault characteristic frequency band, the bearing fault can be diagnosed automatically according to the preset correlation coefficient criterion. Some experimental results were also used to indicate the diagnostic accuracy of the proposed method.

As a timely condition-monitoring method, head-worn displays are being increasingly used in aviation. However, the interface design characteristics that mainly affect head-worn display applications have not been fully investigated. Zhang et al. [6] examined the effects of several important interface design characteristics, such as the distance between calibration lines and the layouts of horizontal and vertical scale belts, on the user preferences and task performances of different head-worn or head-up displays. Their results showed that the participants with head-worn displays performed better than those with head-up displays. The layouts of horizontal and vertical scale belts have a great influence on the task performance and preferences of users. It seems that the users generally preferred interface design characteristics that could yield an optimal performance. These results may facilitate the optimal design of usable head-worn-display technology.

If an early bearing fault is detected, the preventive maintenance of rotating equipment can sustain the high up-time. However, early fault detection is not easily conducted because it is difficult to implement non-intrusive inspections for heavy machinery. As one useful, non-intrusive inspection, machine learning algorithms based on the vibration data from the machine can be helpful. In recent years, machine learning algorithms have been successively used for the early fault detection of different rotational bearings. Petrauskiene et al. [7] presented a fault diagnosis method based on the image classification of different fault patterns for bearings. They used a method of color recurrence plots by using the nonlinear embedding of vibration signals in the delay coordinate space with variable time lags for the feature extraction of the image classification. The database of extracted features of the vibration signals of bearings in the form of color recurrence plots is built to perform deep-learning-based image classification. They used some computational experiments and test data from the Case Western Reserve University to validate the accuracy of the fault classification.

Failure mode diagnosis is the first step in the life prediction of bearings, which should be helpful for protecting the rotors in machinery. Kuo et al. [8] conducted a vibration test, conversion, and analysis of the signals, using characteristic extraction and machine learning with neural networks to obtain the failure mode diagnosis of a rotor-bearing system. In their method, time domain signals were converted into frequency domain signals to analyze the dominant frequency between different sensors. They developed two characteristic extraction methods based on a principal components analysis (PCA) and wavelet packet decomposition (WPD) for an optimization analysis, which were named PCA-WPD and WPD-PCA. They used those two methods to extract the features, which were defined as inputs to long short-term memory networks for classification and training, in order to monitor the bearing status, including the healthy, unbalanced, misaligned, and impact loads.

As a major failure type for thin-walled structures, buckling could be caused by external hydrostatic pressure. Traditional metal materials, including aluminum alloy, titanium alloy, and high-strength steel, are always used to produce submersible pressure shell structures. Due to its limited space and weight, the submarine structure needs materials with a high mechanical performance that are lightweight. Because fiber composite materials have a high stiffness and strength, shells made of fiber composite materials are widely used for external hydrostatic pressure. Moreover, composite materials can improve the structures’ corrosion resistance. Shen et al. [9] used the finite element method to study the buckling of a filament-wound composite cylindrical shell under external pressure. The buckling behavior and pressure under external hydrostatic pressure were predicted. The buckling modes from the simulations and experiments were compared. It seemed that the shell’s axial stiffness was larger than the circumferential stiffness. The strains of the fixed-end and free-end metal control sleeves were analyzed too. It was found that the boundary conditions had a significant influence on the strain response of the control sleeves.

The traffic density and vehicle loads on highways increase with increments in transportation capacity. This may mean that a number of current highway bridges cannot meet the needs of transportation. Moreover, the deterioration of a lot of bridges is very prominent. Thus, an accurate prediction of the performance of highway bridge superstructures is critical for their effective maintenance, because superstructures, including their decks, are an important budget factor for transportation agencies. The safety status prediction and real-time health monitoring of current bridge structures are very useful for small- and medium-span bridge structures. Duan et al. [10] presented a method based on the survival analysis theory to evaluate the service performance of bridge superstructures. The method can assist in the daily repair and maintenance of small and medium bridges. They investigated the influences of the upper general structure, upper load-bearing structure, deck paving, bearings, frequency ratio, and expansion joints on the deterioration of bridge superstructures. It was found that the first-order vibration frequency of the bridge could be identified by the built-in acceleration sensor of a mobile phone. However, the low sensitivity and high output noise may cause an inaccurate diagnosis of the higher-order frequencies. The upper general structure, upper load-bearing members, frequency ratio, deck pavement, and bearing are related to changes in the technical condition level of bridge superstructures.

When a component exceeds its working life or failure, changes in the parameter drift in a system should be caused, which could be affected by the physical properties of the components’ materials, operating environment, and working principle. For the gyroscope in a satellite, if its drift parameters are accurately detected at any time, the drift could be obtained; then, related measures could be used to reduce accidents. Zhang et al. [11] presented an optimal control method for a stochastic system with multiple inputs and multiple outputs by considering the observation noise, external disturbance, and mixed parameter drift. The method can identify and track the drift parameters in a finite time. It can also drive a system to move towards the desired trajectory. The innovation and weight factors were used to produce the dual control algorithm. In this strategy, the analytic solution for the control law could be received. Their algorithm can obtain a better compromise between estimation and optimization. The effectiveness of the algorithm was verified by numerical experiments in two different cases. They also used two different numerical cases to validate their algorithm.

Flexible structures are used in multiple-joint human arms, robotics, aircraft vertical tails, various kinds of manipulators, and gantry crane systems, etc. Due to their thin structural characteristics, vibration problems in flexible arms can be easily caused, which can greatly affect their operation accuracy. To reduce the flexible arm, smart materials are widely applied. Li et al. [12] conducted a test system using smart materials with hysteresis characteristics for nonlinear vibration control, which was an interactive actuator–sensor–controller combination. They designed an integral compensator using an estimation mechanism to reduce the displacements of the flexible arms. The vibration displacements of the flexible arms with and without the integral compensators were compared. They used some experimental cases to verify the proposed interactive actuation vibration control method. It seems that the vibration displacements of the flexible arm could become almost zero within less time and with a lower input power, compared to a traditional controller.

The microvibrations from solar sails, flywheels, and cryocoolers should affect space payloads. The precision of scientific experiments could be limited by these microvibrations. The suppression of microvibrations based on vibration isolation systems is important. Qian et al. [13] proposed an active vibration isolation controller to reduce the effect of vibrations on the variations in cavity length and improve the frequency stability of ultrastable lasers. They designed a state-differential feedback controller with a linear quadratic regulator. They also added a disturbance observer to predict the source noise. They conducted some experiments based on an active vibration isolation system to validate the feasibility and performance of the designed controller. It was found that the accelerations in the axis directions could be suppressed in the low-frequency band within 200 Hz. It seems that the comprehensive vibration may meet the requirements of an ultrastable laser.

Touchscreens’ suitability for human–computer interactions in manned/unmanned aerial vehicle cooperative missions remains uncertain, especially when in time-sensitive conditions with variations under difficult levels. To determine the feasibility of touchscreen applications, Xue et al. [14] introduced a combination of performance and perceptual load measures to divide errors into disposition, undetected, and miscalculation errors. This could explore specific error mechanisms. It could also set up typical manned/unmanned aerial vehicle cooperative human–computer interaction tasks and the antecedent features for potential factors. They conducted some experiments to validate the proposed method. It was found that a higher task difficulty could cause a worse performance and a greater perceived workload by the participants. Their results may support the possibility of using touchscreens in manned/unmanned aerial vehicle cooperative missions.

In recent years, civil aviation companies have tended to adopt aircraft designs with larger aspect ratios to reduce their induced drag, achieve speed breakthroughs, and reduce fuel consumption. However, this design sacrifices the aircraft’s stability and produces flight vibrations [1]. Compared to conventional designs, slender aircraft have greater wave resistance and are subject to increased reaction forces. Therefore, as civil aircraft are improved, more pronounced and sustained vibrations occur [2,3]. Corresponding to the fatigue theory of engineering mechanics, elastic vibrations in flexible aircraft can reduce their service life and may even damage their mechanical structures at certain specific frequencies, leading to more severe effects [3].

Slender aircraft have greater wave resistance, which are also subject to increased reaction forces. Thus, when civil aircraft are improved, more pronounced and sustained vibrations occur. Elastic vibrations in flexible aircraft can reduce their service life, which may damage their mechanical structures at certain specific frequencies. Liu et al. [15] presented an active disturbance rejection controller (ADRC) to suppress the aeroelastic vibrations of a flexible aircraft. They built a vibration model considering the first elastic mode of the flexible aircraft. This ADRC included a simple structure, which can provide better control than the traditional proportional-integral-derivative (PID) control theory. Moreover, it was easier to translate from theory to practice compared to other modern control theories. It found that the ADRC suppressed aircraft elastic vibrations better than the PID controllers and that the closed-loop system was robust in the face of dynamic parameters.

## 3. Conclusions, Outlook, and Acknowledgments

In this Special Issue, many outstanding world scientists provided different contributions from many industry fields. The guest editors think this Special Issue may provide some academic contributions to advanced vibration-based fault diagnosis and vibration control methods for industry applications.

The authors pay special appreciation to all 38 authors of the articles, as well as all professional and diligent reviewers.
